# On the Plakobranchidae (Gastropoda, Sacoglossa) from soft sediment habitats of Koh Tao, Gulf of Thailand, with descriptions of two new species

**DOI:** 10.3897/zookeys.969.52941

**Published:** 2020-09-17

**Authors:** Rahul Mehrotra, Manuel Caballer Gutiérrez, Chad M. Scott, Spencer Arnold, Coline Monchanin, Suchana Chavanich

**Affiliations:** 1 Reef Biology Research Group, Department of Marine Science, Faculty of Science, Chulalongkorn University, Bangkok 10330, Thailand; 2 Conservation Diver, 7321 Timber Trail Road, Evergreen, Colorado, 80439, USA; 3 The American University of Paris, Department of Computer Science Math and Environmental Science, 6 rue du Colonel Combes, 75007 Paris, France; 4 Muséum national d’Histoire naturelle, 55 rue de Buffon, 75005 Paris, France; 5 Research Center on Animal Cognition (CRCA), Center for Integrative Biology (CBI); CNRS, University Paul Sabatier – Toulouse III, France; 6 Center of Excellence for Marine Biotechnology, Department of Marine Science, Faculty of Science, Chulalongkorn University, Bangkok 10330, Thailand

**Keywords:** biodiversity exploration, cryptic species, *
Elysia
*, Heterobranchia, *
Plakobranchus
*

## Abstract

Research in recent years have provided rapid advances in biogeographic and taxonomic documentation of sea slugs around the world. However, efforts are lacking in surveying most coastlines and habitats in South-East Asia. Recent studies from the Gulf of Thailand have indicated that a wealth of unexplored sea slug diversity and ecology may be gained from an investigation of soft sediment habitats beyond the reef slopes. Additionally, the waters of Koh Tao have been found to host regionally high levels of sea slug diversity with several species awaiting taxonomic clarification. In this work the initial findings of an expanded survey effort from the waters around Koh Tao are provided, with the identity of two soft sediment-associated sacoglossan species in the family Plakobranchidae being investigated. By integrating morphological and molecular analyses, the species *Plakobranchus noctisstellatus***sp. nov.** and *Elysia aowthai***sp. nov.** are described and species complexes surrounding *Plakobranchus ocellatus* van Hasselt, 1824 and *Elysia japonica* Eliot, 1913 are discussed. The topics of morphological variability and the cryptic species problem are also discussed.

## Introduction

In Thailand, most of the research carried out on sacoglossan sea slugs occurred in the last 30 years, after the description of *Cylindrobulla phuketi* Jensen, 1989. Since then, a total of 21 species have been recorded in Thailand (Andaman Sea and Gulf of Thailand combined), with seven of these being described within Thai waters. These include *C. phuketi*, *Elysia siamensis* Swennen, 1998, and *E. bangtawaensis* Swennen, 1998; *Gascoignella nukuli* Swennen, 2001 and *Swennenia jabae* (Swennen 2001); *Costasiella coronata* Swennen, 2007, and *Ercolania halophilae* Jensen, Kohnert, Bendell & Schrödl, 2014. Of the 21 species, six are recorded only from the Andaman Sea in western Thailand, ten exclusively within the Gulf of Thailand, and the remaining are known either from both seas or their precise collection location is unknown.

A recent inventory of traditional ‘opisthobranch’ sea slugs from the island of Koh Tao, Gulf of Thailand, yielded 87 species and documented 32 new records for the Gulf of Thailand region (25 of which for all Thai waters), particularly highlighting the remarkable role of soft sediment habitats in the local sea slug diversity (Mehrotra and Scott 2016). It was found that 37% of all species recorded around Koh Tao were recorded exclusively from the soft sediment habitats outside of the coral reef, including the majority of species found at the time to be as yet undescribed, and rarely observed by the local recreational diving community. As part of this study, two species of *Plakobranchus* van Hasselt, 1824 were identified from the island’s waters, *Plakobranchus ocellatus* van Hasselt, 1824 and *Plakobranchus ianthobaptus* Gould, 1852, later amended to Plakobranchus cf. ocellatus and Plakobranchus cf. papua respectively (Mehrotra et al. 2019). Molecular evidence has shown that the species historically identified as *P. ocellatus* represents a complex of multiple species (Krug et al. 2013) and thus at present the recently described *Plakobranchus papua*Meyers-Muñoz et al., 2016 is the only species that is considered taxonomically stable (see discussion).

The analysis of soft sediment sea slugs of the island also included a species of the genus *Elysia* Risso, 1818 that was found to bear characteristics corresponding to different species and was recorded as *Elysia* sp. Later, during trials on the ingestive capabilities of scleractinian corals, the same species was referred to as Elysia cf. japonica (Mehrotra et al. 2019), due to the resemblance with the species described by Eliot (1913) from Japan. Since the original description of *Elysia japonica* Eliot, 1913, numerous authors have described other morphologically similar species, based on a variety of characters which were missing in the description of *E. japonica*, whose type appears to be lost (Trowbridge et al. 2011). The taxonomic uncertainty of these species has been raised numerous times over the decades (see Jensen 1985; Rudman 2001; Trowbridge et al. 2011; Takano et al. 2013 and others), with *Elysia abei* Baba, 1955, *E. amakusana* Baba, 1955, and *E. furvacauda* Burn, 1958, all at one point or another being suggested as synonyms of *E. japonica*.

Such cases as those of *Plakobranchus ocellatus* and *Elysia japonica* are part of a rapidly growing subset of taxonomic murkiness referred to as the ‘cryptic species’ problem. These are often characterised by discrepancies between morphological and molecular analyses (Korshunova et al. 2019), with increasing access to molecular technologies allowing for growing documentation of genetic divergence within groups with overlapping external morphologies (Jörger and Schrödl 2013; Korshunova et al. 2019). Incidences of molecular analyses revealing previously undocumented species complexes or challenging historic synonymisations are abundant within the family Plakobranchidae Gray, 1840 (i.e., Krug et al. 2013, 2016). It is therefore apparent that comprehensive and integrated analyses are needed in the description of such species, including aspects of internal and external morphology, genetics, as well as ecology.

Since the initial findings recorded by Mehrotra and Scott (2016), more extensive surveys on sea slug biodiversity and ecology have been carried out, with a focus on the soft sediment habitats. In this work we conducted a molecular analysis to clarify the status of the species in the genus *Plakobranchus* from Koh Tao, as a result describing a new species exclusive to deeper soft sediment habitats from the island. We also provide an integrated molecular analysis of a species belonging to the complex surrounding *E. japonica*, discussing its long and complex taxonomic history, and providing detailed morphological information for the species inhabiting Thai waters, which is described as a new taxon based on genetic and geographical criteria.

## Materials and methods

### Sampling and anatomical studies

Specimens of *Plakobranchus* and specimens of *Elysia* resembling *E. japonica* were collected by SCUBA diving at Koh Tao, Thailand, on soft sediment habitats at depths ranging from 1 to 25 meters and photographed in-situ with an Olympus TG-4 camera with an underwater housing. When specimens were collected, 95% ethanol was used for preservation for both molecular and morphological analysis. Anatomical studies were performed using an Olympus SZX16 stereomicroscope, which was also used for the preparation of glycerine slides for light microscopy of radula, eyes, and penial apparatus. A TESCAN-VEGA-II-LSU scanning electron microscope (**SEM**) belonging to the Plateau Technique de Microsocopie Electronique of the Muséum national d’Histoire naturelle, Paris, France (**MNHN**) was used for this study. The SEM observations were performed on dried and gold-coated samples with an accelerating voltage of 15 kV. Images were taken with an Everhart-Thornley detector. Type specimens are deposited at the MNHN. No permissions were required for sample collection and all permissions for analyses were acquired through Chulalongkorn University, Thailand. Some paratypes of the described species are also stored at the Reef Biology Research Group (**RBRG**) in the Department of Marine Science, Chulalongkorn University.

### DNA extraction, amplification, and sequencing

Tissue was taken from the ventral region of the foot of each specimen and DNA extracted using Quiagen DNeasy Tissue Kits. Primer sequences for partial sequences of cytochrome c oxidase subunit I (COI) were sourced from Folmer et al. (1994) using pairs LCO1490 (5’-GGTCAACAAATCATAAAGATATTGG-3’) and HC02198 (5’-TAAACTTCAGGGTGACCAAAAAATCA-3’). Partial sequences of the 16S rRNA region were amplified using the forward primer 16Sar-L (5’-CGCCTGTTTATCAAAAACAT-3’) from Palumbi et al. (1991) and reverse primer 16s-xH (5’-CCGGTYTGAAMYYAGATCACGTAGG3’) from Mehrotra et al. (2020). Primers for the Histone 3 region were taken from Colgan et al. (2000) using the primers H3F (5’-ATGGCTCGTACCAAGCAGACVGC-3’) and H3R (5’-ATATCCTTRGGCATRATRGTGAC-3’). PCR was carried out using BioRads MJ Mini™ Personal Thermal Cycler with a reaction volume of 20 μl. PCR protocol for the COI region was as follows: an initial denaturing step at 94 °C for 3 minutes; 40 cycles of denaturing at 94 °C for 30 seconds, annealing at 45 °C for 30 seconds, an extension at 72 °C for 1 minute, followed by a final extension at 72 °C for 10 minutes. PCR protocol for the partial 16S region and the nuclear H3 region was: an initial denaturing step at 94 °C for 3 minutes; 40 cycles of denaturing at 94 °C for 30 seconds, annealing at 53 °C for 30 seconds, an extension at 72 °C for 1 minute, followed by a final extension at 72 °C for 10 minutes. The same protocol was used for both 16S primer combinations. Electrophoresis was carried out using 0.5% TBE agarose gel. Purified aliquots were sent to Macrogen (Macrogen Sequencing Services: http://dna.macrogen.com/eng/) for sequencing.

### Sequence and phylogenetic analyses

Available sequences for multiple species (Table 1) of *Plakobranchus* and of multiple species of *Elysia*, including numerous sequences from those species which are cryptic with *Elysia japonica*, were used for phylogenetic analysis. Sequences were sourced from published material (specifically Bass 2006; Krug et al. 2013; Takano et al. 2013; Krug et al. 2015, 2016) and GenBank (NCBI). All sequence metadata such as sample identifier and location were verified based on published material as primary quality control, with GenBank metadata used if sequences were unpublished or unverified. All sequences were aligned and edited using BioEdit 7.2.5 (Hall 1999) and then reviewed manually. Primers were trimmed from the resulting alignment. The pairwise distances for COI, 16S, and H3 genes were calculated using the Kimura 2 parameters model implemented in MEGA 5.1 (Tamura et al. 2011). We applied a liberal 7% (COI) threshold to suggest a possible species delimitation criterion; however, only a closer morphological analysis of more specimens will yield a clearer picture of an appropriate molecular stringency in the discussed clades. Automatic Barcode Gap Discovery (**ABGD**) analyses (Puillandre et al. 2012) were conducted on the complete COI dataset without the outgroup. Three different ABGD analyses were performed to delineate species within the COI dataset. Each analysis was run using a different nucleotide substitution model, JC69, K80 2.0, and Simple Distance, with the settings Pmin = 0.001, Pmax = 0.1, Steps = 10, X = 1.5, Nb bins = 20. Phylogenetic analyses were carried out by using both Maximum Likelihood (**ML**), and Bayesian Inference (**BI**) methods. Analysis for each was conducted on sequences from the COI region independently, followed by an analysis of concatenated sequences of COI and 16S and H3 regions. Optimum evolutionary models were selected using the model test feature within MEGA 7.0.14 (Kumar et al. 2016). The optimum model used for analyses of COI and concatenated sequences was GTR+G+I. Analysis was conducted with 1000 bootstrap replicates and random starting trees. All sequences were additionally analysed using Bayesian Inference via MrBayes 3.2 (Ronquist et al. 2012). Analysis was conducted with 50,000,000 generations and four chains with Markov chains being sampled every 1000 generations. The first 25% generations were removed as burn-in with the rest being used to produce the 50% consensus tree.

**Table 1.** Sequences used in this study. New sequences are in bold, the remainder were obtained from GenBank.

Species	Location	COI	16S	H3
*Elysia abei*	Japan	KM086374	JN819137	JN819171
*Elysia abei*	Japan	KC573711	–	–
*Elysia abei*	Japan	KC573712	–	–
*Elysia abei*	Japan	KC573713	–	–
*Elysia abei*	–	JN819115	–	–
*Elysia abei*	Japan	AB758953	AB759022	–
*Elysia abei*	Japan	AB758954	AB759023	–
*Elysia abei*	Japan	AB758955	AB759024	–
*Elysia amakusana*	Japan	AB758956	AB759025	–
*Elysia amakusana*	Australia	GQ996686	EU140851	–
*Elysia asbecki*	Vanuatu	KM086360	KM204200	KM040808
*Elysia bangtawaensis*	Thailand	KM086375	KM204224	KM040826
*Elysia chlorotica*	Massachusetts, USA	KM086377	KM204226	JN819183
*Elysia diomedea*	Panama	KM086379	KM204228	KM040830
*Elysia furvacauda*	Australia	KM086369	KM204218	KM040821
*Elysia hamatani*	Japan	JN819110	JN819143	JN819177
***Elysia aowthai* sp. nov.**	**Gulf of Thailand**	**MK835779**	**MK835763**	**MK835771**
***Elysia aowthai* sp. nov.**	**Gulf of Thailand**	**MK835780**	**MK835764**	**MK835772**
***Elysia aowthai* sp. nov.**	**Gulf of Thailand**	**MK835781**	**MK835765**	**MK835773**
*Elysia marginata*	Guam	JN819100	–	–
*Elysia obtusa*	Japan	KM086387	KM204236	KM040840
*Elysia rufescens*	Japan	AB758961	–	–
*Elysia singaporensis*	Singapore	KM086398	KM204249	KM040847
*Elysia* sp. 5	Hawaii	JN819113	JN819138	JN819172
Elysia cf. japonica	Japan	AB758952	–	–
Elysia cf. japonica	Guam	DQ471255	DQ480176	DQ534772
*Plakobranchus ocellatus*	Australia	GQ996680	–	–
*Plakobranchus ocellatus*	Philippines	JX272720	–	–
*Plakobranchus ocellatus*	Philippines	JX272696	–	–
*Plakobranchus ocellatus*	Philippines	JX272695	–	–
*Plakobranchus ocellatus*	Philippines	JX272688	–	–
*Plakobranchus ocellatus* (black)	Japan	KC573718	–	–
*Plakobranchus ocellatus* (black)	Japan	AB758971	–	–
*Plakobranchus ocellatus* (blue)	Japan	KC573714	–	–
*Plakobranchus ocellatus* (blue)	Japan	AB758968	–	–
*Plakobranchus ocellatus* (blue)	Guam	KC573717	KM204279	KM040891
*Plakobranchus ocellatus* (purple)	Japan	KC573727	KM204280	KC597161
*Plakobranchus ocellatus* (purple)	Japan	KC573726	–	–
*Plakobranchus ocellatus* (purple)	Japan	AB758969	–	–
*Plakobranchus* sp. (aff. purple)	French Polynesia	KC573729	KM204276	KM040890
*Plakobranchus* sp. (aff. purple)	Guam	KC573730	–	–
*Plakobranchus* sp. (spotless)	Japan	KC573731	KM204283	KM040893
*Plakobranchus ocellatus*	Guam	HM187634	KM204284	KM040894
*Plakobranchus ocellatus* (white)	Japan	AB758970	–	–
*Plakobranchus ocellatus* (white)	Japan	KC573719	–	–
*Plakobranchus ocellatus* (white)	Guam	KC573722	–	–
*Plakobranchus ocellatus* (white)	Thailand	KC573723	–	–
*Plakobranchus ocellatus* (white)	Australia	KC573725	–	–
***Plakobranchus ocellatus***	**Gulf of Thailand**	**MK835784**	**MK835768**	**MK835776**
***Plakobranchus papua***	**Gulf of Thailand**	**MK835785**	**MK835769**	**MK835777**
***Plakobranchus papua***	**Gulf of Thailand**	**MK835786**	**MK835770**	**MK835778**
*Plakobranchus papua*	Indonesia	KC573732	KM204281	KC597163
*Plakobranchus papua*	Indonesia	KU934191	–	–
*Plakobranchus papua*	Indonesia	KU934192	–	–
*Plakobranchus papua*	Indonesia	KU934193	–	–
***Plakobranchus noctisstellatus* sp. nov.**	**Gulf of Thailand**	**MK835782**	**MK835766**	**MK835774**
***Plakobranchus noctisstellatus* sp. nov.**	**Gulf of Thailand**	**MK835783**	**MK835767**	**MK835775**
*Plakobranchus* (aff. sp. 1)	Papua New Guinea	KC573734	KM204277	KC597165
*Plakobranchus* (sp. 2)	Philippines	KC573736	KM204282	KM040892
*Plakobranchus* sp.	Hawaii	KY012787	–	–
*Plakobranchus* sp.	Hawaii	KY012788	–	–
*Plakobranchus* sp.	Hawaii	KC573738	–	–
*Thuridilla albopustulosa*	Guam	KM086443	KM204302	KM040916
*Thuridilla gracilis*	Guam	KM086444	KM204304	KM040917
*Thuridilla hopei*	Italy	KC573743	KM204305	KC597170
*Thuridilla livida*	Malaysia	KC573745	KM204307	KC597172
*Thuridilla splendens*	Japan	KM086445	KM204310	KM040920
*Costasiella coronata*	Hong Kong	KJ610067	KJ610027	KJ610054
*Costasiella usagi*	Guam	KJ610071	KJ610031	KJ610058
*Cyerce elegans*	Vanuatu	KM086353	KM204193	KM040801

## Results

### Phylogenetic analyses

**Figure 1.**
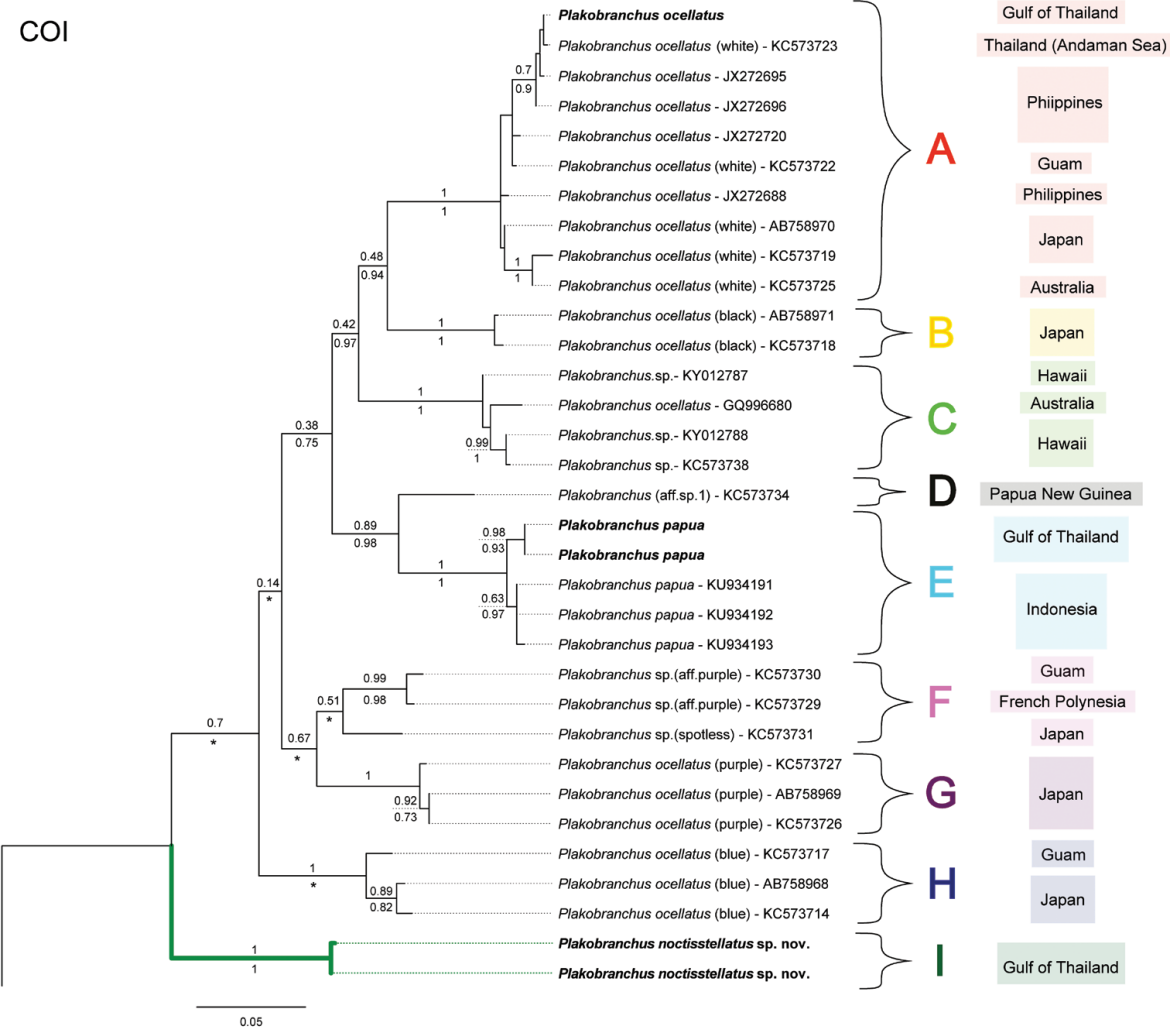
Phylogenetic hypothesis of *Plakobranchus* based on COI sequences. Bootstrap values from ML shown above branch and posterior probability (PP) values below branch. Sequences obtained in this study in bold. Missing PP values, or those that support branch placements that deviate from the ML tree are denoted with an asterisk * and are due to the discrepancy between ML and BI topologies (see Suppl. material 1). Tree rooted to *Costasiella coronata* (not shown).

All molecular analyses (Fig. 1) consistently place *Plakobranchus noctisstellatus* sp. nov. as not only distinct from all other sequenced species and morphs belonging to the genus *Plakobranchus* with strong support, but also basal to the genus in both stand-alone COI and concatenated phylogenies (100% PP and BS values). Uncorrected pairwise distances for each gene for *Plakobranchus noctisstellatus* were found to have a minimum distance of 12%, 10%, and 7% for COI, 16S, and H3 respectively when compared to its congeners (Table 2). Distances for COI were also calculated for other available *Plakobranchus* sequences which were then informally separated into clades with a liberal minimum distance threshold of 7%. Concatenated phylogenies of the combined sequences also rendered *Plakobranchus* as monophyletic with high support (100% PP and 99% BS). Initial approaches of ABGD analyses consistently indicated nine partitions (Fig. 1, Table 2); however, recursive analyses using JC69 and K80 models indicated a tenth partition separating *Plakobranchus papua* from Koh Tao as sister to those from the type locality. In all analyses, *Plakobranchus noctisstellatus* sp. nov. was distinct from all other clades of the genus.

**Table 2.** Distance values within and between clades corresponding to the COI phylogeny of *Plakobranchus*.

Clade	In-clade min	In-clade max	Out-clade min
A	0%	4%	10%
B	–	1%	9%
C	0%	2%	10%
D	–	–	7%
E	0%	2%	7%
F	1%	6%	8%
G	0%	1%	7%
H	1%	3%	9%
I	0%	0%	12%

**Figure 2.**
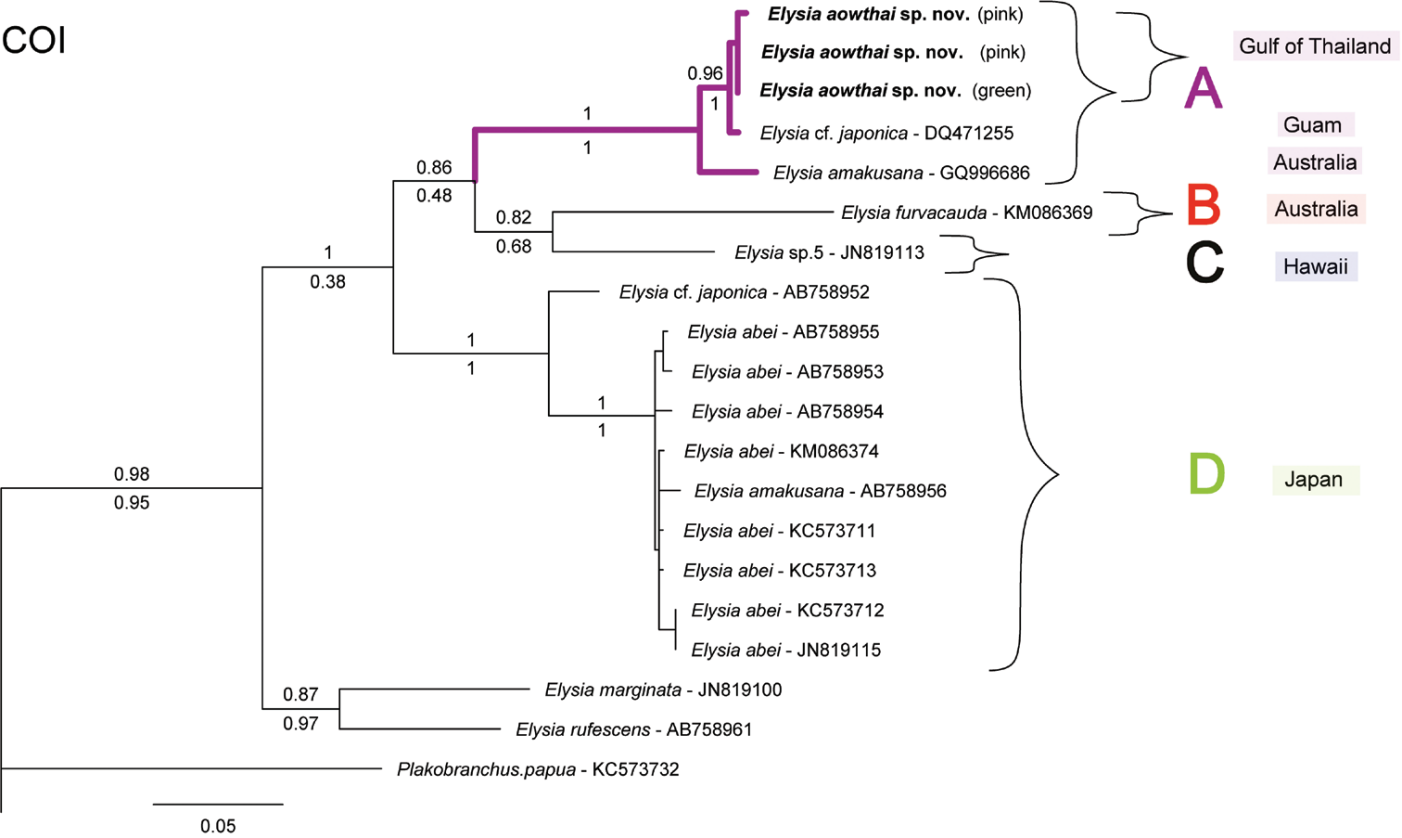
Phylogenetic hypothesis for the *Elysia japonica* complex based on COI sequences. Sequences obtained in this study in bold. Bootstrap values from ML shown above branches and PP values from BI below branches. Tree rooted to *Costasiella coronata* (not shown).

Strong support was also found for *Elysia aowthai* sp. nov. as a distinct species in both stand-alone COI (100% PP and 96% BS) and concatenated phylogenies (100% PP and BS values). Distances for *E. aowthai* sp. nov. were calculated within the clade (Clade A in Fig. 2), and minimum distances for species outside the clade for each gene. The maximum intra-clade distance for each gene was found to be 6% (COI), 7% (16S), and 0% (H3), with the minimum distance for specimens outside the clade being 11% (COI), 9% (16S), and 6% (H3) (Table 3). All three ABGD analyses with all *Elysia* sequences used outputted consistent results, revealing 13 stable different partitions within the dataset via the initial approach. However, all three also suggested a 14^th^ partition via the recursive approach. The *E. japonica* complex divided into four clades (Fig. 2, Table 3) in the initial approach, with the recursive approach separating Elysia cf. japonica (from Japan) into a fifth distinct part. Nonetheless, in all analyses the *Elysia aowthai* sp. nov. clade (Clade A) was well supported as distinct from all others, including the two specimens identified as E. cf. japonica from Guam (Bass 2006) and *E. amakusana* from Australia (Wägele et al. 2010).

**Figure 3.**
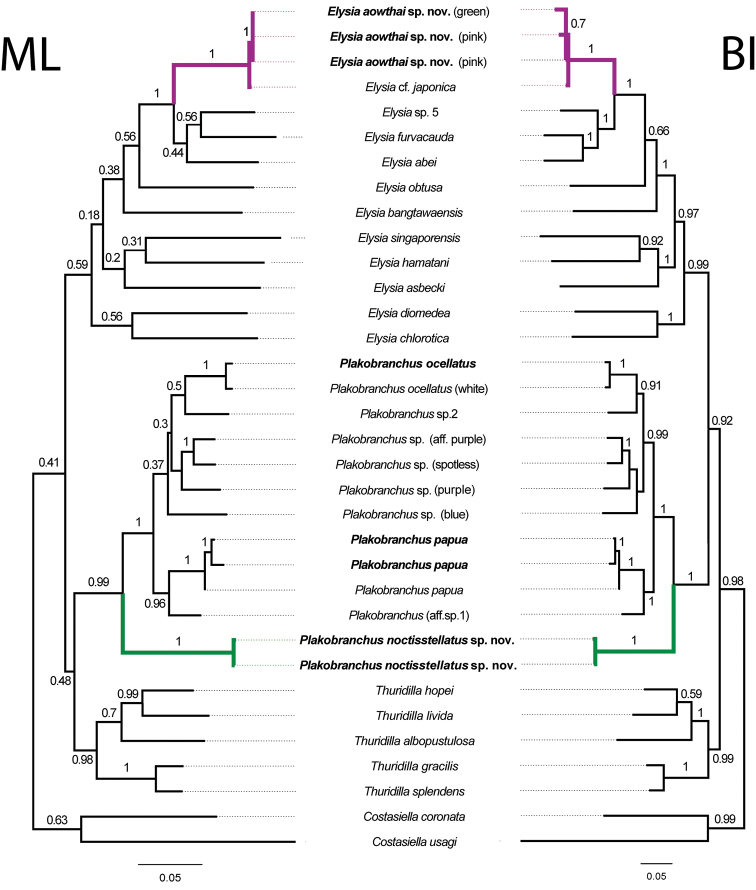
Phylogenetic hypotheses of Plakobranchidae based on concatenated sequences of COI, 16S, and H3 regions. Sequences obtained in this study in bold. Bootstrap values from ML topology (left) aligned with PP values from BI topology (right).

Phylogenies documented here largely agree with others (Bass and Karl 2006; Krug et al. 2013; Takano et al. 2013; Krug et al. 2016) with the family Plakobranchidae being separated into two clades. The first clade includes the genera *Plakobranchus* and *Thuridilla*, which form monophyletic sister subclades with strong support, and the species in *Elysia* formed a second well-supported clade (Fig. 3). Likewise, the genus-specific analysis using the COI gene for *Plakobranchus* also agrees with the previous observations (Krug et al. 2013, 2016) that there is indeed an extensive complex of species needing formal description. A similar case is seen for the *Elysia japonica*/*abei*/*amakusana*/*furvacauda*/*aowthai* complex with numerous cases of mistaken identities and at least four distinct species delimited under analysis of the COI gene (Fig. 2, Table 3).

**Table 3.** Distance values within and between clades corresponding to the COI phylogeny of the *Elysia japonica* complex.

**Clade**	**In-clade min**	**In-clade max**	**Out-clade min**
A	0%	4%	12%
B	–	–	11%
C	–	–	11%
D	1%	6%	12%

### Systematics

#### Class Gastropoda Cuvier, 1795

**Subclass Heterobranchia Burmeister, 1837**

**Superorder Panpulmonata Jörger, Stöger, Kano, Fukuda, Knebelsberger & Schrödl, 2010**

**Order Sacoglossa Ihering, 1876**

**Suborder Plakobranchacea Gray, 1840**

**Superfamily Plakobranchoidea Gray, 1840**

**Family Plakobranchidae Rang, 1829**

##### Genus Plakobranchus

Taxon classificationAnimaliaSacoglossaPlakobranchidae

van Hasselt, 1824

7CF83E4C-8090-525B-ADAE-7561A428571F

###### Diagnosis.

Body wide, dorsoventrally flattened with broad parapodial flaps folding along dorsal midline, tail truncate. Head broad and flattened with a pair of small, raised, median eyes and rolled, laterally originating, rhinophores. Parapodial lamellae with digestive gland branches and dorsal haemolymph sinuses. Anus anterodorsal and penis armed with curved stylet. Radular teeth denticulate.

##### Plakobranchus ocellatus

Taxon classificationAnimaliaSacoglossaPlakobranchidae

van Hasselt, 1824

A42C1226-4E2E-5FDD-B55B-973081B12A79

**Figure 4.**
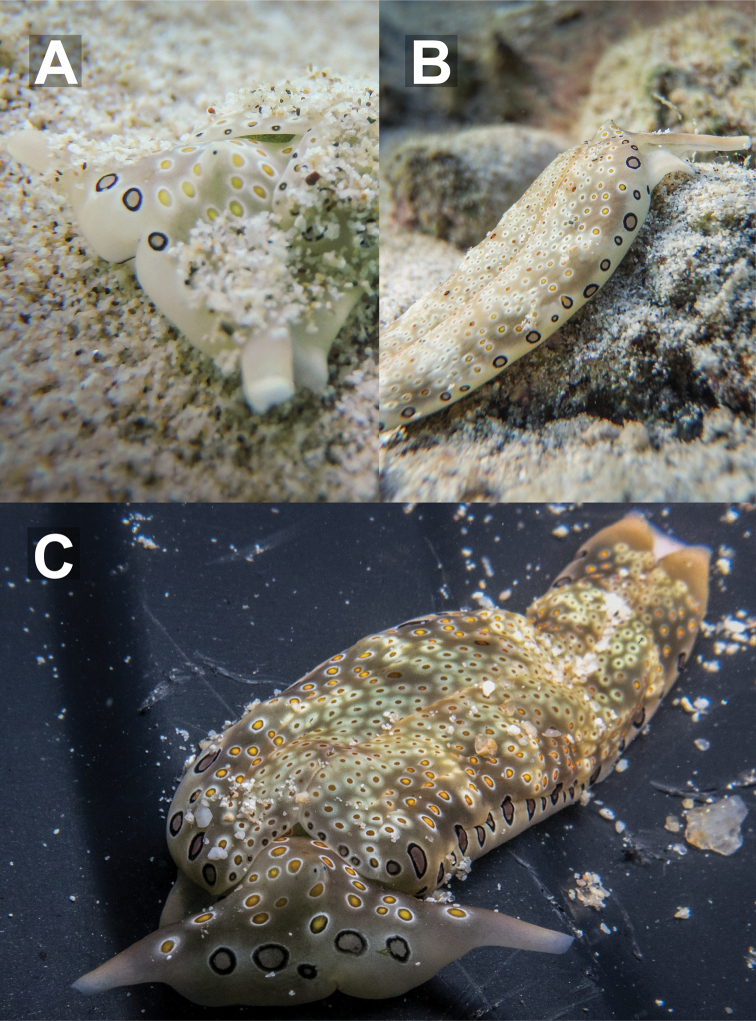
Living specimens of *Plakobranchus ocellatus* from Koh Tao. **A, B** close-up of head with retracted rhinophores and dorsolateral view, 25 mm **C** sequenced specimen, 32 mm.

Figure 4 , Suppl. material 2

Plakobranchus ocellatus : Christa et al. 2013: 560, fig. 1A, D (Luminau, Guam; Australia)
Plakobranchus ocellatus (white): Krug et al. 2013: (Andaman Sea, Thailand; Japan; Australia; Guam)
Plakobranchus ocellatus (white): Takano et al. 2013: fig. 3K (Japan)
? Plakobranchus ocellatus*s. s.*: Meyers-Muñoz et al. 2016: 91, Table 2
Plakobranchus ocellatus : Tanamura and Hirose 2016: 5, fig. 3A (Ryuku Archipelago, Japan)
Plakobranchus sp. 6: Gosliner et al. 2018: 434 (Philippines)
Plakobranchus ocellatus : Yonow and Jensen 2018: 20, fig. 5I (Bohol, Philippines)

###### Material examined.

Three specimens 25–32 mm Chalok Bay, 10°3'44.77"N, 99°49'30.35"E, Koh Tao, Thailand.

###### Supplementary observations.

More than 100 individuals, ranging in size 5 mm–45 mm, observed in regular diving surveys between 2012 and 2019, Chalok Bay, 10°3'44.77"N, 99°49'30.35"E, Shark Bay 10°3'39.75"N, 99°50'4.43"E; Tao Tong 10°3'58.13"N, 99°49'4.76"E; Sai Nuan 10°4'43.24"N, 99°48'48.51"E; Twins 10°7'1.93"N, 99°48'44.26"E; Hin Wong Bay 10°6'12.30"N, 99°50'58.63"E, Koh Tao, Thailand; not collected.

###### Description.

Length alive up to 45 mm. Background colour pale yellowish white to pale brown, covered in ocelli, increasing in size laterally from parapodial margins. Dorsal ocelli small, brown or brown with yellow centres, surrounded by a diffuse ring of white. Dorso-laterally, ocelli that have a yellow centre and a brown ring followed by a white diffuse ring are also found on the head between rhinophores. Lateral ocelli large with a grey centre, thick black ring followed by thin diffuse white ring; 3–7 of these are also found on the anteriormost part of the head. Tips of rhinophores translucent bluish grey, not easily visible upon retraction, followed by white diffusing to the same pale colour as the dorsum. Rhinophores rolled, long, extending laterally from the head, curved like bull horns.

Parapodial margins translucent when opened, with yellowish white spots visible along the edge beneath the tissue surface. Internally parapodial ridges thick, bright green, with no visible spots. Eyes black, very close together, placed centrally on the head, held raised above the rest of the head when crawling. Oral prominences globose with a very fine black line on the edge of the upper lip. Anterior foot corners and tail edged in the same translucent bluish grey as rhinophore tips. Male genital opening located behind the right rhinophore, above the foot corner, in front of the anterior part of the parapodia. Penis translucent white when extended in living specimens. Foot sole white with numerous black spots throughout.

###### Ecology.

From shallow soft sediments to sandy areas along the reef edge. Rarely in deeper soft sediment habitats beyond the reef edge. Depth 0.5–11 m.

###### Distribution.

*Plakobranchus ocellatussensu lato* is currently considered widespread across the Indo-Pacific including Kenya, Zanzibar, the Red Sea, Maldives, Seychelles, Reunion (Yonow 2012), India (Sheeja and Padma Kumar 2014), the Philippines (Christa et al. 2013), Indonesia (Eisenbarth et al. 2018; Yonow and Jensen 2018), Japan (Maeda et al. 2012), Australia, Papua New Guinea (Yonow and Jensen 2018), Guam (Wägele et al. 2011), Vanuatu (Krug et al. 2013), Hawaii (Wade and Sherwood 2016), Tanzania, Madagascar, Malaysia and Palau (Gosliner et al. 2008). Specimens considered as *P. ocellatus* have been previously recorded from the Andaman and Gulf waters of Thailand (Jensen 1992; Nabhitabhata 2009).

###### Remarks.

The genus *Plakobranchus* has undergone dramatic changes over the past two centuries with more than a dozen species being described in the 1800’s and all being synonymised with the type taxon *Plakobranchus ocellatus* by numerous authors in later years (e.g., Bergh 1887; Jensen 1992). *Plakobranchus ocellatus* was described based on blue spots with yellow centres seen dorsally and laterally on a pale ground colour and some information on the pericardial and reproductive anatomy. The species has regularly been recognised/identified by numerous authors based on many of these external characteristics (Rao 1960; Mercier and Hamel 2005; Wägele et al. 2010; Maeda et al. 2012; Sheeja and Padma Kumar 2014; Mehrotra and Scott 2016; Wade and Sherwood 2016). Recent research suggests that the dramatic synonymisation of species under the name *P. ocellatus* may have been premature, with molecular evidence suggesting at least ten independent clades within the complex of *P. ocellatus* (Krug et al. 2013, 2016). This supports the findings of previous authors who have observed different morphs of *P. ocellatus* which appeared to be externally distinguishable based on the general colouration and the distribution of the ocelli or the spots on the dorsal or ventral surface (Ono 2005; Trowbridge et al. 2011; Krug et al. 2013; Yonow and Jensen 2018). While Krug et al. (2013) were able to provide evidence that multiple species historically identified as *P. ocellatus* are likely different, no images nor detailed morphological descriptions or comparisons were provided. It is assumed, however, that all morphotypes identified therein bear some external resemblance to *P. ocellatussensu stricto*, in particular a white or pale ground dorsal colour.

##### Plakobranchus papua

Taxon classificationAnimaliaSacoglossaPlakobranchidae

Meyers-Muñoz & van der Velde, 2016

AB6788C8-5249-522C-AFAC-CE84ABAD7986

**Figure 5.**
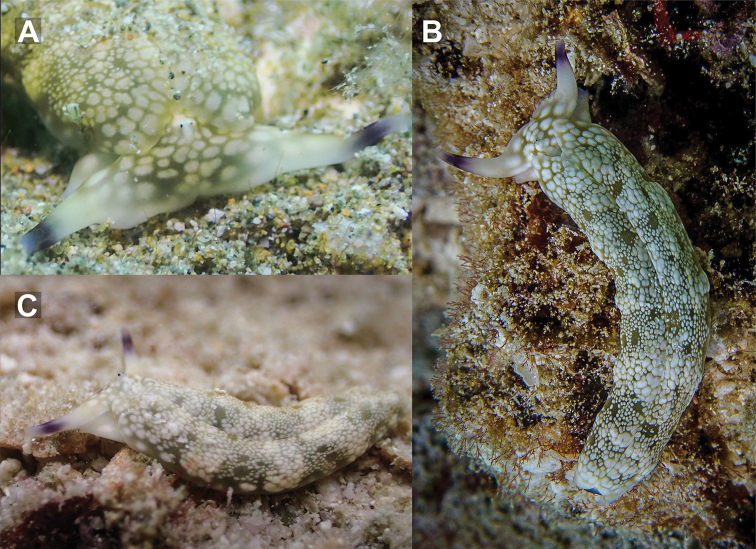
Living specimens of *Plakobranchus papua* from Koh Tao **A** close-up of head, 28 mm specimen **B** dorsal view, 30 mm (photograph by Pau Urgell Plaza) **C** dorsolateral view of sequenced specimen, 27 mm.

Figure 5 , Suppl. material 3

**Figure 6.**
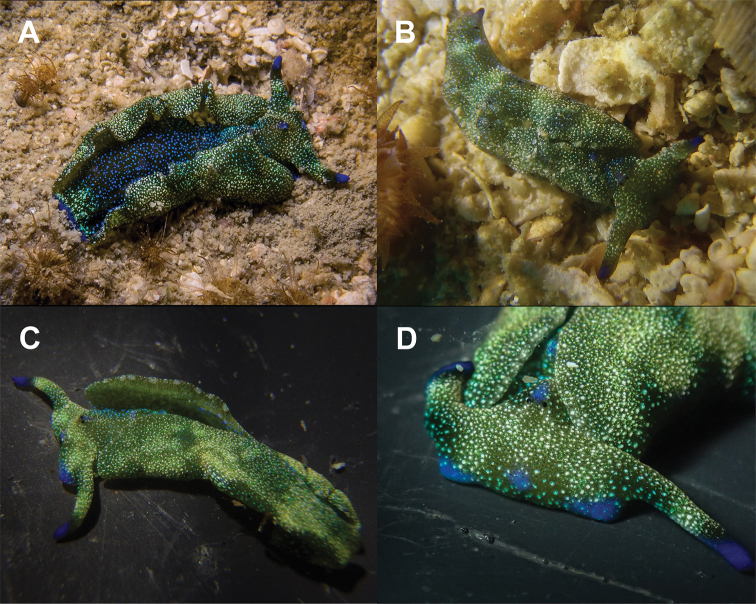
Living specimens of *Plakobranchus noctisstellatus* sp. nov. from Koh Tao. **A** 21 mm (photograph by Pau Urgell Plaza) **B** 15 mm **C, D** (paratype) 31 mm.

Plakobranchus ocellatus : Coleman 2008: 88 (Milne Bay, Papua New Guinea; Uepi Island, Solomon Islands)
Plakobranchus sp. 1: Gosliner et al. 2008: 94 (Philippines; Indonesia; Papua New Guinea)
Plakobranchus papua Meyers-Muñoz & van der Velde in Meyers-Muñoz et al. 2016: 80–88, figs 2–7a (West Papua, Indonesia).
Plakobranchus papua : Gosliner et al. 2018: 434 (Indonesia)
Plakobranchus cf. papua : Yonow and Jensen 2018: 21, 27–30, figs 6C, D, 12, 13C, D, 14C, D (Ambon, Indonesia).

###### Material examined.

Three specimens 19–30 mm Sai Nuan, 10°4'43.24"N, 99°48'48.51"E, Koh Tao, Thailand.

###### Supplementary observations.

27 individuals, ranging in size 12 mm–35 mm, observed in regular diving surveys between 2012 and 2019, Chalok Bay, 10°3'44.77"N, 99°49'30.35"E, Shark Bay 10°3'39.75"N, 99°50'4.43"E; Tao Tong 10°3'58.13"N, 99°49'4.76"E; Sai Nuan 10°4'43.24"N, 99°48'48.51"E, Koh Tao, Thailand; not collected.

###### Description.

Length alive up to 35 mm. Background colour varies from pale yellow to an almost translucent greyish white, lacking the brownish/ochre background in the original description (Meyers-Muñoz et al. 2016: fig. 3a–c) and other Indonesian material (Yonow and Jensen 2018). Dorsally covered in white dots of varying sizes from the anterior-most part of the head to posterior edge of parapodia. These are also visible dorsally on the blunt anterior foot corners. There are prominent spots on the dorsum where no white dots are present and only the background colour is present. Rhinophores are translucent white with a diffuse band of deep blue to dark purple before the translucent tips. Rhinophores rolled, long, extending laterally from the head, curved like bull horns.

Parapodial margin with distinct white rod-like spots, almost identical to but more continuous than those in the Indonesian specimens (Meyers-Muñoz et al. 2016: fig. 3a; Yonow and Jensen 2018). Internal parapodial ridges bright to dark green, slightly thinner than in *P. ocellatus*, with no visible spots. Eyes black, very close together, centrally on the head, held slightly raised above the rest of the head when crawling. Tail tip dark blueish purple, almost black, diffusing to white anteriorly. Male genital opening located behind the right rhinophore, above the foot corner, in front of the anterior part of the parapodia. Penis transparent to translucent with a bluish white tip when extended in living specimens, penile bulb and ducts clearly visible inside. Foot sole completely white with no spots, posteriorly tinged in deep purple visible dorsally on the tail tip.

###### Ecology.

Abundant in shallow soft sediment habitats and among the corals and soft sediments of the reef edge. Uncommon, but present in dense coral reef habitats; rare in deeper soft sediment habitats outside the coral reef. Has been observed being ingested naturally by the scleractinian coral *Pleuractis paumotensis* but is mostly considered unpalatable by such corals (Mehrotra et al. 2015, 2019).

###### Distribution.

Known only from the Philippines, Malaysia, Indonesia, and Papua New Guinea (Meyers-Muñoz et al. 2016; Yonow and Jensen 2018). Known from Gulf waters of Thailand (Mehrotra et al. 2015).

###### Remarks.

This species has previously been referred to, erroneously, as *Plakobranchus ianthobaptus* Gould, 1852 (Mehrotra and Scott 2016) and Plakobranchus cf. papua (Mehrotra et al. 2019). Externally, specimens of *P. papua* from Koh Tao differ from the original description of the species. Specifically, the background pigmentation of the parapodia varies from pale yellow to an almost translucent greyish white, lacking the ochre background tinge and the yellow discontinuous line in the border of the parapodia known to date for the species. Furthermore, the specimens of *P. papua* in the original description show scattered white dots of varying sizes on the surface of the parapodia, whilst in specimens from Koh Tao they almost completely cover the surface. The rhinophores and tail from Koh Tao specimens are deep blue to dark purple at the tips (rarely black), rather than almost entirely covered by black pigment as in the specimens from the original description. A very similar variant to that documented from Koh Tao was documented from Ambon, Indonesia, as Plakobranchus cf. papua by Yonow and Jensen (2018: fig. 6C, D). Minor differences between the Ambon specimens and those from Koh Tao are the paler dorsal colouring and more continuous rod-structures along the parapodial margins in Koh Tao specimens. Additionally, the longitudinal white line visible behind the eyes in Ambon specimens appears to be broken up in specimens from West Papua (Meyers-Muñoz et al. 2016: fig. 3A–D) and scattered in Koh Tao specimens (Fig. 5). Molecular data presented here suggest the present specimens as conspecific with *P. papua* and the additional material highlights the external variation in the species.

##### Plakobranchus noctisstellatus sp. nov.

Taxon classificationAnimaliaSacoglossaPlakobranchidae

A53E27B2-F1C7-5805-A0CA-4A48691B14C5

http://zoobank.org/2A0E4A50-5567-4470-86F5-7B21690C9A66

**Figure 7.**
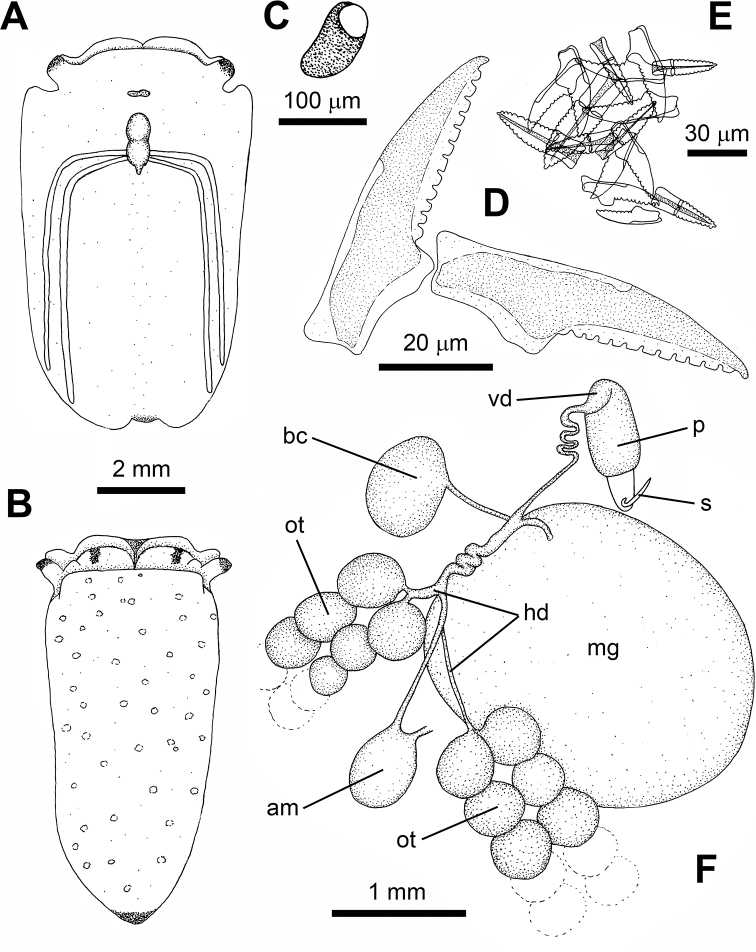
*Plakobranchus noctisstellatus* sp. nov. **A** dorsal view of the animal **B** ventral view of the animal, **C** detail of the eye **D** last teeth of the ascending series and active teeth **E** teeth in the ascus **F** reproductive system. Abbreviations: am – ampulla; bc – bursa copulatrix; hd – hermaphrodite duct; mg – mucus gland; ot – ovotestis, p – penis; s – stylet; vd – vas deferens.

Figures 6 , 7 , 10D–F

Plakobranchus sp.: Coleman 2008: 89 (Thailand; Gorontalo, Indonesia)
Plakobranchus sp. 2: Gosliner et al. 2008: 94 (Vanuatu; Bali, Indonesia); Gosliner et al. 2015: 98 (Papua New Guinea).
Plakobranchus ocellatus var. I: Meyers-Muñoz et al. 2016: 91, Table 2
Plakobranchus cf. ocellatus : Yonow and Jensen 2018: 30 (top of 2^nd^ column), fig. 6E (Bali, Indonesia)

###### Type material.

***Holotype***: adult, 28 mm long (alive), collected in silty sand at 21 m depth, Sai Nuan, 10°4'43.24"N, 99°48'48.51"E, Koh Tao, Thailand, 06 April 2016, deposited in MNHN (IM-2000- 35324). ***Paratype***: adult, 31 mm long (alive), collected in silty sand at 18 m depth, Tao Tong, 10°3'58.13"N, 99°49'4.76"E, Koh Tao, Thailand, 18 March 2017, deposited in MNHN (IM-2000- 35325). Paratype dissected: reproductive system studied, and jaw, radula, and penis mounted for optical microscopy. ***Paratype***: adult, 26 mm long (alive), collected in silty sand at 24 m depth, Tao Tong, 10°3'58.13"N, 99°49'4.76"E, Koh Tao, Thailand, 17 February 2020, deposited in RBRG (PkII-NR011).

###### Supplementary observations.

More than ten individuals, ranging from 10 mm to up to 45 mm, observed in regular diving surveys between 2016 and 2018, Tao Tong 10°3'58.13"N, 99°49'4.76"E; Sai Nuan 10°4'45.02"N, 99°48'45.23"E; Shark Bay 10°3'39.75"N, 99°50'4.43"E; Koh Tao, Thailand, not collected.

###### Description.

Length alive up to 45 mm. Body wide, dorsoventrally flattened with wide parapodial flaps folding along dorsal midline. Background colour bright green to dark green, with scarce black spots, and abundant opaque white spots all over. Some white spots with blue hue, others with yellowish tinge. Five or six prominent black spots similar in size and shape to the eyes found laterally on both sides. Tips of rhinophores and tail are electric blue, followed by a black band, not so evident in the tail. Rhinophores long, rolled, extended from lateral edges of the head, curved like bull horns when crawling. Internal parapodial flaps ridges bright green to dark green, with white and electric blue spots. Eyes black, very close to each other, sometimes with a blue hue between them, located in a groove between rhinophores. Oral prominences globose, with a big black patch on each side, and a very fine, undulating black line on the edge of upper lip. Anterior-upper end of the oral prominences green with white spots and the same electric blue as rhinophore tips. Anterior foot corners in preservation blunt. Foot sole the same green as the dorsal surface with several small iridescent light blue spots. A possible transverse foot groove may be present; however, this was not distinct in living specimens and equally as vague upon preservation. Tail truncated. Dorsal region dark green to black, with big opaque white to electric blue spots (Fig. 6).

***Renopericardial*** prominence composed of two globose, oval lobes (in preservation). Posterior lobe pointed at the end, with pair of major haemolymph sinuses, both perpendicular to its lateral surface and turning at right angles once in the parapodia. Haemolymph sinuses thick, cord-like, white in preservation, longitudinal, parallel to each other (Fig. 7A), not joining together at the ends. Most external haemolymph sinuses shorter. Internal surface of parapodia smooth in preservation, except for the haemolymph sinuses that externally seem to run from the renopericardial prominence. Anal opening at the right anterior side of the pericardium (Fig. 7A).

***Uniserial radula*** (in one 20 mm long specimen) with eight teeth including ghost teeth in ascending row, ten in descending row, 24 in ascus. Teeth triangular, sharp, bearing striations (Fig. 10), and with two cutting edges and 12 or 13 strong and blunt denticles on each of them. First teeth in ascending series 65 μm long, 18 μm high. Active teeth 60 μm long, 18 μm high. Last teeth in descending series 50 μm long, 17 μm high. Teeth in the ascus in varied stages of degradation, 25–66 μm long, packed haphazardly (Fig. 7E).

***Penial bulb*** approximately 1 mm long, below rhinophores, at same level as the eyes, bearing a sharp cuticular stylet. Stylet 280 µm long, hollow, kinked at the base, with the tip like the tip of a hypodermic syringe. Vas deferens connected to penial bulb, curved and strong; medial part thinner and coiled; proximal part straight, over mucus gland, at the end joins a wider conduct. This conduct connected to mucus gland, then coiled and bifurcated to ampulla and to ovotestis. Bursa copulatrix connected to the underside of the central fertilisation area near by the end of the vas deferens (Fig. 7A). Ovotestis grape-shaped, composed of spheres filling the parapodia. Spheres variable in diameter, from 350 to 500 µm. Mucus gland large, globose (Fig. 7F).

###### Biology.

All animals were observed year-round at different locations around the island, exclusively in deeper soft sediment habitats at Koh Tao. Animals found either partially buried in or moving across the open silt and sand dominated habitats beyond the fringing coral reefs of the island. Animals found at depths from 15–25 m with no indication of seasonal variation. No observations made shallower than 15 m depth or in the vicinity of coral reef or reef edge habitats. Not observed to be in association with any particular prey algae, nor any other organism in particular, and as such, its prey remains unknown. While multiple individuals have been recorded in close proximity, mating was never observed, nor egg masses identified.

###### Derivatio nominis.

*Plakobranchus noctisstellatus* from the Latin words *noctis* (night) and *stellatus* (stellate), in reference to the small iridescent blue and green spots hidden under the dark parapodia that each resemble stars at night.

###### Distribution.

*Plakobranchus noctisstellatus* sp. nov. is known from Thailand and has been recorded under different names in Vanuatu, Indonesia, and Papua New Guinea (Gosliner et al. 2008, 2015).

###### Remarks.

*Plakobranchus papua* Meyers-Muñoz and van der Velde, 2016, was described based on morphological and molecular evidence, distinguishing it from *P. ocellatus*. Specimens of *P. papua* are characterised by an ochre body with white non-ocellated spots, black rhinophores and tail, and a foot sole without spots. Additionally, the radular teeth of *P. papua* were described as more ‘arched’ than those shown in descriptions provided of *Plakobranchus ocellatussensu lato.* Molecular evidence in this study sheds some light on the external variation of the species (see Discussion). Meyers-Muñoz et al. (2016) also tackled a visual comparison of multiple *Plakobranchus* varieties that have historically been identified as *Plakobranchus ocellatus*, including a species almost identical and likely corresponding to *Plakobranchus noctisstellatus* sp. nov. being referred to as *Plakobranchus ocellatus* var. I. Meyers-Muñoz et al. (2016, Table 2) also indicate that an illustration of this animal (similar to *Plakobranchus noctisstellatus* sp. nov.) may have been provided by Gould (1852: pl. 26, fig. 407a–c, as *Placobranchus ianthobaptus*) but Yonow and Jensen (2018) refuted this and stated that there was no resemblance between the two. We concur, in that *Placobranchus ianthobaptus* can in fact be distinguished externally from *P. noctisstellatus* sp. nov. by its pale brown dorsal ground colour.

*Plakobranchus noctisstellatus* sp. nov. is easily distinguished both externally and internally from both *P. papua* and *P. ocellatus*. Externally *P. noctisstellatus* sp. nov. is most easily separated from its congeners and the variants of *P. ocellatus* by its vibrant green ground colour and electric blue rhinophores and tail, and notably the dense collections of white and electric blue spots under the parapodial flaps. The presence of non-ocellated black spots, prominent laterally, and iridescent blue spots on the green foot sole also make separation between species easy. The present study suggests that rhinophore colouration in *P. papua* may vary from black (as described) to deep purple with white tips such as those from Koh Tao; however, this variation remains distinct from the electric blue tips and black band found in *P. noctisstellatus* sp. nov. While little information on rhinophore colouration was provided for *P. ocellatus* upon description, the original illustrations of the species by van Hasselt (1824) do show rhinophores that are entirely white or cream, much like specimens of *Plakobranchus ocellatus* from Koh Tao. The range in rhinophore colouration of different morphs and synonyms of *P. ocellatus* vary from white to purple tips or bands and black lines along rhinophore edges, all of which differ from *P. noctisstellatus* sp. nov. While *Placobranchus guttatus* Stimpson, 1855 was described as bearing a very similar dorsal colour of dark olive, the species was also described as having green ocellated spots with white rings, which are absent from *P. noctisstellatus* sp. nov.

The reproductive system of *P. ocellatus* is distinguished from that of *P. noctisstellatus* sp. nov. by having two bursae copulatrix, a bilobed mucus gland (not rounded), and much smaller ovotestis (follicles). Compared to that of *P. papua*, the reproductive system of *P. noctisstellatus* sp. nov. shows a smaller stylet and much larger acorn-shaped penial bulb, lacking the groove observed in *P. papua*; a curved vas deferens that rapidly decreases in thickness to become convoluted and connects to the ovotestis, the bursa copulatrix and the ampulla, that Meyers-Muñoz et al. (2016) identify as a second bursa copulatrix; the ovotestis, which Meyers-Muñoz et al. (2016) call follicles are much larger in *P. noctisstellatus* sp. nov.

The number of radular teeth seen in *P. noctisstellatus* appears similar to those of its congeners with eight in the ascending limb (eight in *P. papua* and 7–11 in *P. ocellatuss. l.*) and ten in the descending limb (seven in *P. papua* and 7–9 in *P. ocellatuss. l.*). The number of denticles per radular tooth seen in *P. noctisstellatus* sp. nov. (12 or 13) fits within the range of species described thus far (10–14 in *P. papua* and 10–13 in *P. ocellatuss. l.*); however, the overall shape of the teeth appears to show some variation among the species. The teeth of *P. noctisstellatus* sp. nov. have striations and are more curved than those of *P. papua*, which in turn was described to have teeth that appeared to be more arched than those of *P. ocellatus* by Bergh (1873) and Jensen (1997). Additionally, the denticles seen in the SEM image of *P. ocellatus* by Jensen (1992) are more prominent and proportionally larger than those seen in *P. noctisstellatus* sp. nov., which seem to be more uniform and regular in appearance. It should be noted that van Hasselt (1824) provided no information on the radula of *P. ocellatus* in the original description. Additionally, there appears to be significant plasticity in radular morphology based on diet in numerous species of Sacoglossa (Jensen 1993), but conclusive evidence pertaining to the diet of *P. noctisstellatus* sp. nov. could not be obtained and thus requires further investigation. At Koh Tao, there are significant differences in the ecology of *P. noctisstellatus* when compared to *P. papua* and *P. ocellatus*. While the former exists exclusively in the deeper soft sediment habitats of the island, the latter species are mostly observed among coral reefs and reef flats closer to shore and the soft sediments therein.

Many of these points and observations were also made in the most recent documentation of *P. noctisstellatus* sp. nov. (as *Plakobranchus* sp.), where Yonow and Jensen (2018) also provided a tabulation comparing the morphology of all species thus far synonymised with *P. ocellatus*. Including their sightings, most observations recorded for specimens most likely being *P. noctisstellatus* sp. nov. are from Indonesia, with Gosliner et al. (2008) also recording the species from Vanuatu; therefore, specimens from the Gulf of Thailand represent the western-most range of the species so far.

##### Genus Elysia

Taxon classificationAnimaliaSacoglossaPlakobranchidae

Risso, 1818

E4F370FC-DFDF-5EEF-A9FF-81CCE912E11C

###### Diagnosis.

Body smooth to papillate, with parapodia that may cover much of the dorsal surface; however, parapodia can be highly variable and may not be held close to the body. Head differentiated from the body with eyes behind dorsal rhinophores. Dorsal sinuses usually branched, anus anterodorsal, reproductive system pseudo-diaulic or triaulic, penis normally unarmed although a hollow apical stylet may be present. Radular teeth blade-shaped ranging from denticulate to smooth.

##### Elysia aowthai sp. nov.

Taxon classificationAnimaliaSacoglossaPlakobranchidae

81D627CF-B6F2-5DAC-B7D6-176DAF029B44

http://zoobank.org/2605D690-FD7A-4830-8CD6-5474C050DB26

**Figure 8.**
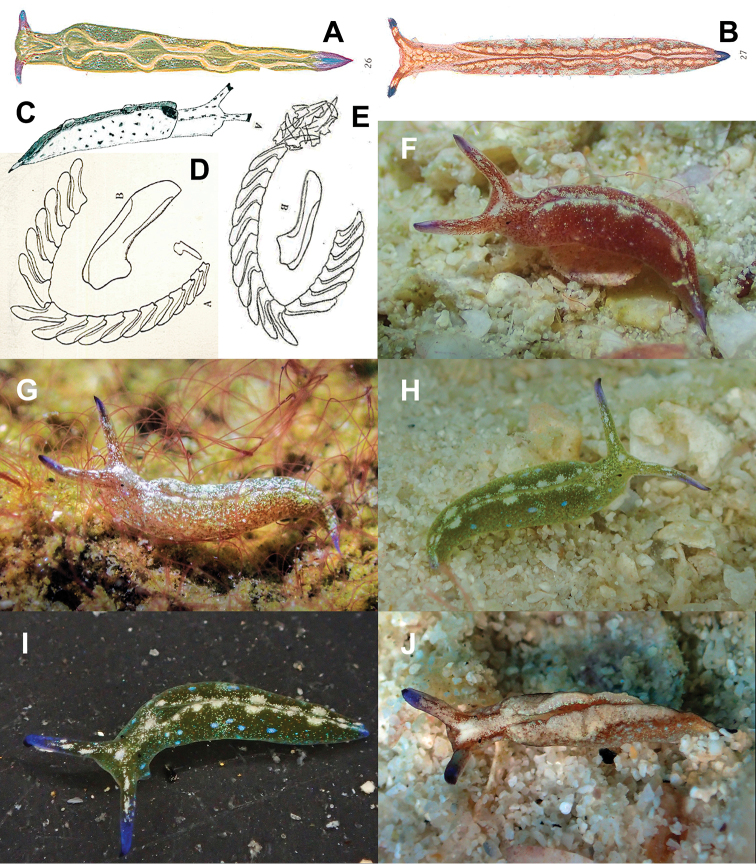
Illustrations and images of specimens belong to the *Elysia japonica* complex **A–E** illustrations taken from the original descriptions of species belonging to the *Elysia japonica* complex **A, D***Elysia abei* Baba, 1955 **B***Elysia amakusana* Baba, 1955 **C***Elysia furvacauda* Burn, 1958 **E** illustration of radula of *Elysia japonica* Eliot, 1913 by Baba (1949)**F–J***Elysia aowthai* sp. nov. showing variation in colouration, 12 mm (**F**), 9 mm (**G**), 11 mm (**H, I**), 8 mm (**J**).

**Figure 9.**
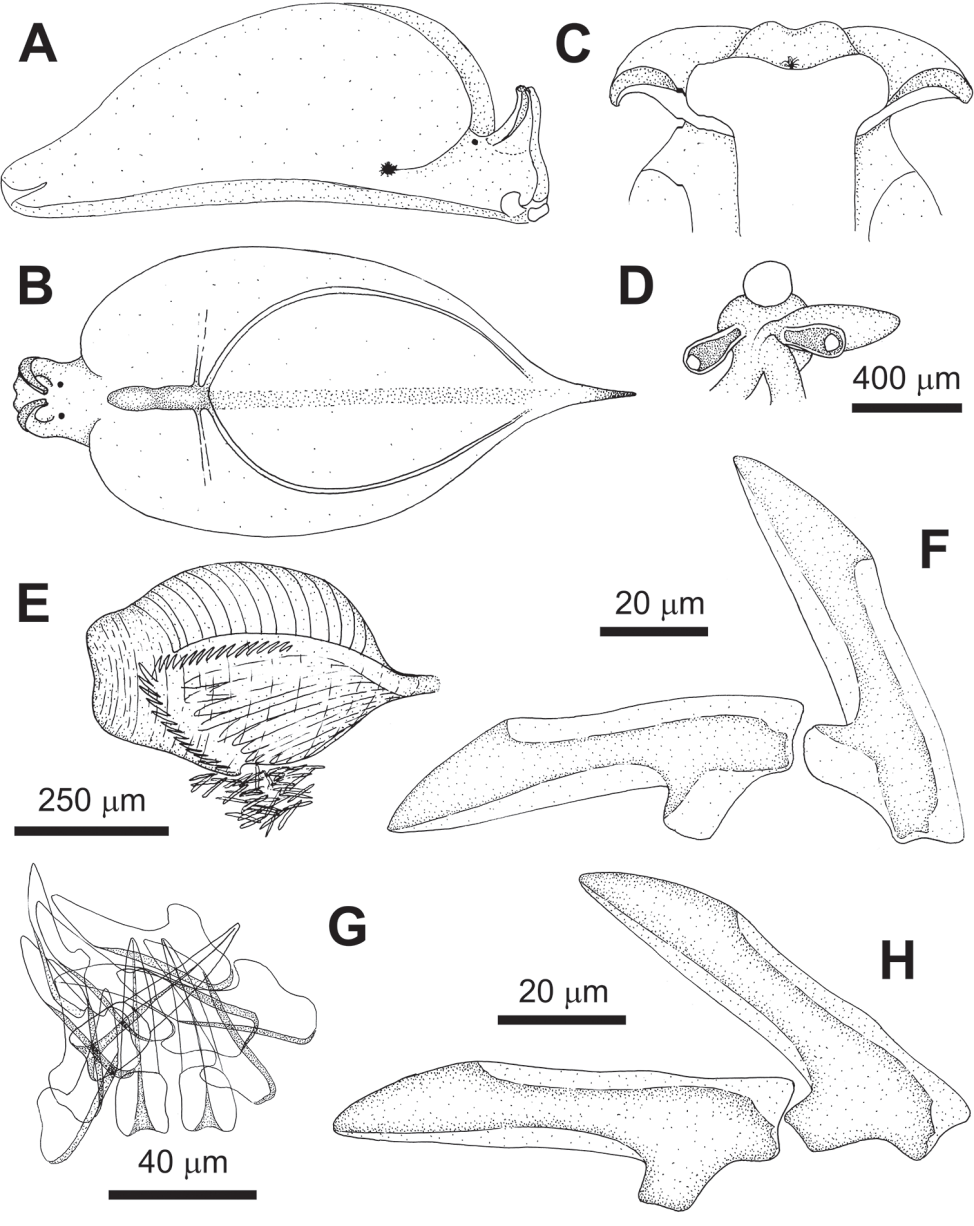
*Elysia aowthai* sp. nov., Koh Tao Thailand **A–F** 16 mm alive **A** right side view, the black dot at the end of the parapodia represents the vaginal opening **B** dorsal view, the shaded area probably being the same as that mentioned by Eliot (1913: 47) **C** ventral view with a detail of the head **D** detail of the eyes **E** buccal bulb **F** last teeth of the ascending series and active teeth **G, H** 10 mm alive **G** ascus **H** last teeth of the ascending series and active teeth.

**Figure 10.**
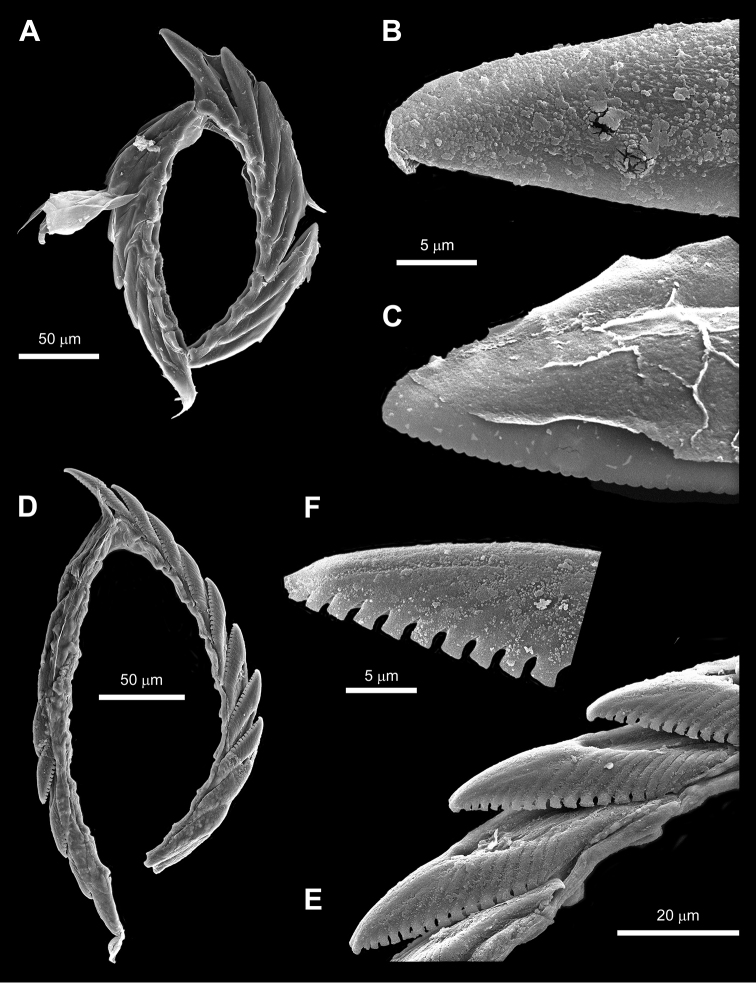
SEM images of radulae of *Elysia aowthai* sp. nov. and *Plakobranchus noctisstellatus* sp. nov. **A–C***Elysia aowthai* sp. nov., Koh Tao, Thailand, 15 mm alive **A** general view of the radula without the ascus **B** active teeth from the radula with smooth cutting edge **C** sixth teeth of the descending series with denticulated cutting edge **D–F***Plakobranchus noctisstellatus* sp. nov. Koh Tao, Thailand, 21 mm alive **D** general view of the radula without the ascus **E** lateral view of the descending series **F** detail of the cutting edge of a tooth.

**Figure 11.**
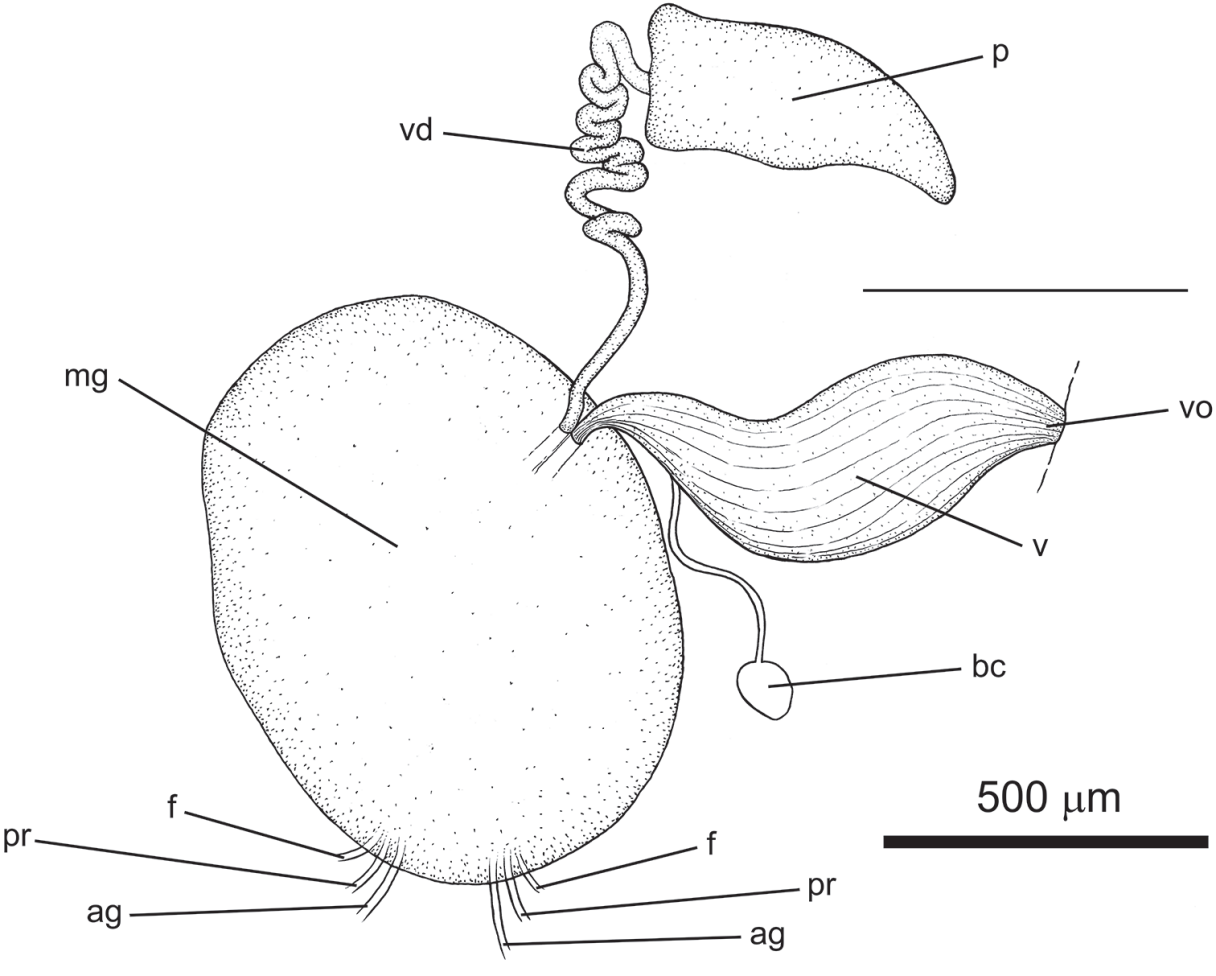
Genitalia and reproductive system of *Elysia aowthai* sp. nov., Koh Tao, Thailand, 15 mm alive. Abbreviations: ag – to the albumen gland; bc – bursa copulatrix; f – to the follicles; mg – mucus gland; p – penis; pr – to the prostate; v – vagina; vd – vas deferens; vo – vaginal opening.

Figures 8F–J , 9 , 10A–C , 11

Elysia cf. japonica : Bass 2006 (Guam)
Elysia amakusana : Wägele et al. 2010 (Lizard Island, Australia)
Elysia sp. 1: Mehrotra and Scott 2016: fig. 2B, C (Koh Tao, Thailand)
Elysia cf. japonica : Mehrotra et al. 2019: fig. 1C (Koh Tao, Thailand)

###### Type material.

***Holotype***: adult, 14 mm long (alive), collected from soft sediment habitats at 12 m depth, Leuk Bay (type locality) 10°4'15.23"N, 99°50'32.86"E, Koh Tao, Thailand, 20 December 2015, deposited in MNHN (IM-2000- 35326). ***Paratype***: adult, 16 mm long (alive), collected from soft sediment habitats at 16 m depth, Tao Tong 10°3'58.13"N, 99°49'4.76"E, Koh Tao, Thailand, 19 June 2017, deposited in MNHN (IM-2000- 35327). Paratype dissected: reproductive system studied, and jaw, radula, and penis mounted for optical microscopy.

***Paratype***: adult, 14 mm long (alive), collected in silty sand at 25 m depth, Tao Tong, 10°3'58.13"N, 99°49'4.76"E, Koh Tao, Thailand, 17 February 2020, deposited in RBRG (EcjIV-NR012).

###### Supplementary observations.

More than 100 individuals, ranging in size 5 mm–16 mm, observed in regular diving surveys between 2012 and 2019, Leuk Bay 10°4'15.23"N, 99°50'32.86"E; Suan Olan Artificial Reef 10°4'6.70"N, 99°50'26.29"E; Shark Bay 10°3'39.75"N, 99°50'4.43"E; Tao Tong 10°3'58.13"N, 99°49'4.76"E; Sai Nuan 10°4'43.24"N, 99°48'48.51"E; Twins 10°7'1.93"N, 99°48'44.26"E; Hin Wong Bay 10°6'12.30"N, 99°50'58.63"E; Laem Tien 10°5'19.13"N, 99°51'17.64"E Koh Tao, Thailand; not collected.

###### Description.

Length alive up to 16 mm. Body translucent white, with tips of rhinophores deep blue to purple, fading to the base and lacking tubules of digestive gland. Opaque white specks all over the body, concentrated on the edge of parapodia, renopericardial prominence, and dorsal surface of head and rhinophores. Digestive gland variable from reddish brown to light green, forming a characteristic reticulated pattern of thin tributaries. Eyes black, conspicuous, comma-shaped, behind rhinophores, bearing 75 µm diameter lentil-shaped crystalline lenses. Rhinophores long, pointed at the tip, with groove along entire length. Lateral groove on right side from anterior border of right parapodium to foot. Foot’s transversal groove at same height as end of lateral groove, very subtle, almost invisible, dividing foot in two. Anterior foot corners slightly extended (in preservation), bluntly pointed. Body and parapodia in some specimens with sparse small papillae which disappear upon preservation. Tail pointed, extending beyond parapodia (Fig. 8).

***Renopericardial*** prominence long and narrow, oval anteriorly, slightly constricted in the middle, and straight posteriorly. Posterior end with two pairs of major dorsal sinuses: first short, perpendicular, fading at the middle of the parapodium; second long, oblique, curved, orientated towards but not reaching edge of parapodium, ending before the tail. Area between second pair of dorsal sinuses appearing translucent in preserved specimens (Fig. 9B) and probably corresponding to the ‘smooth trench’ mentioned by Eliot (1913: 47) describing *E. japonica* (KR Jensen, pers. comm.). Renal pore at anterior right side of pericardium. Anus in groove separating the right parapodium from the neck (Fig. 9).

***Buccal bulb*** 500 μm long (in three 10, 15, and 16 mm long specimens). Uniserial radula with seven teeth including ghost teeth in ascending row, 8–11 in descending row. Discarded teeth driven out of buccal bulb through a short tube; 12–30 in the ascus, varied in size, some broken, stacked in several groups, some loose. Teeth narrow, blade-shaped. Cutting edges smooth or finely denticulated. Denticulation difficult to observe with the light microscope and random (Fig. 10). First teeth in ascending series 72–76 μm long, 22–25 μm high. Active teeth 71–80 μm long, 27–30 μm high. Last teeth in descending series 50–76 μm long, 20–29 μm high.

***Penis*** unarmed, pseudo-conical with a narrow and curved end (chilli-shaped), 500 μm long. Vas deferens long and convoluted, passing through the mucus gland. Male genital opening below right rhinophore, next to right eye. Vagina fusiform, narrow at the extremes, passing through the mucus gland. Bursa copulatrix connected to the vagina. Mucus gland quasi-oval, translucent, 900–1000 μm long, located under the cardiac area. Ducts from the follicles, prostate, and albumen gland coming from the parapodia meet in the mucus gland, but the connections between them are unclear (Fig. 11).

**Biology.** All animals were observed year-round at different locations around the island, exclusively in deeper soft sediment habitats at Koh Tao. Animals were always found moving along the surface of the sediments, rarely stationary and with no specific ecological relationship with any other species. Not observed to be in association with any particular prey algae, but often found in the vicinity of a currently unknown filamentous red algae, which may contribute to the reddish colouration seen in most specimens. Specimens with green, brown, purple, and unpigmented digestive glands are likely due to some degree of variability in prey; however, further investigations are required. No observations were made shallower than 10 m depth or in the vicinity of coral reef or reef edge habitats, deeper observations up to 24 m.

###### Derivatio nominis.

*Elysia aowthai* in reference to the type locality of the species in the Gulf of Thailand, ‘Aow Thai’ in the local vernacular language, and to honour the local populations that speak it.

###### Distribution.

Gulf of Thailand (present study), Guam (as E. cf. japonica, Bass 2006), and Australia (as *E. amakusana*, Wägele et al. 2010) based on our molecular analyses.

###### Remarks.

The general appearance of *Elysia aowthai* sp. nov. places it within the *E. japonica* species complex. The problem with this complex has its origins in the incomplete description of *E. japonica* itself, but also in the provenance of the type material and the subsequent specimens captured, sometimes associated with descriptions and illustrations and sometimes not. *Elysia japonica* was described based on preserved specimens, which makes the external anatomy and living colouration very difficult to untangle, collected from an unknown location in Japan. The complete description by Eliot (1913) is as follows:

“*18 specimens. Locality – unknown. The largest is about 20 mm long and the wings are moderately ample. In two specimens which were dissected, the radula was found to contain 5 teeth in the ascending row, 15 in the descending and about 20 more various sizes lying in a heap. The structure and the shape of the teeth is as usual in the genus. No denticles are to be seen. I think that this form is probably a new species distinguished by the following characters*:

*1) Colour: In all specimens, the rhinophores and the tip of the tail are conspicuously black or dark brown. Otherwise the colour is uniform and in the best preserved specimens is yellowish brown. The wings have no coloured borders and the head and the pericardium are of the same colour as the dorsal surface.*

*2) The arrangement of the dorsal surface. This is similar in all specimens and I have not seen it in any other species. The pericardium is not ovate but is constricted in the middle. Its length is greater than its breadth but it is short in comparison with the length of the whole animal. The dorsal ridges which run into it are very distinct and the two hindmost, which run backwards towards the tail, are parallel to one another and enclose an area which is differentiated from the back and forms a smooth trench.*”

The first illustration showing the external anatomy of a specimen assumed to be *Elysia japonica* was provided by Baba (1949), with a description of the species. However, this was later changed in a supplement to the previous paper wherein the same specimen (Baba 1949: plate IX, fig. 27) was reidentified and claimed as a new species (Baba 1955), *Elysia amakusana* Baba, 1955, based exclusively on the presence of finely denticulated teeth, which are (theoretically) not present in *E. japonica*. Also, in the same supplement, Baba described *Elysia abei* Baba, 1955, which also has finely denticulated teeth but is differentiated by being green in colour with fine orange-red spots (Baba 1949: plate VIII, fig. 26; Baba 1955). Afterwards, however, Baba determined all three species as valid in a summarised inventory of *Elysia* species from Japan (Baba 1957). A year later, *Elysia furvacauda* Burn, 1958 was described from Australia (Burn 1958), based entirely on the external anatomy (therefore lacking any mention of radular detail), as a red-brown animal with small blue spots and rhinophores, and tail tipped in black. The type material for this species was reported as lost by Jensen (1985), but it is actually deposited in Museums Victoria Collections (MVC 2020, registration number F19467) (KR Jensen, pers. comm.).

Subsequently, Marcus (1980) and later Jensen (1985) both pointed out the similarity in radular teeth between *E. amakusana* and *E. abei*, with Jensen providing a description of three different colour morphs of *E. japonica* from Hong Kong, a comprehensive analysis of the taxonomic confusion to date, and concluding that *E. amakusana* and *E. abei* are junior synonyms of *E. japonica*. Importantly, Jensen showed that the morphology of specimens of three different external colourations was remarkably similar and that radular teeth have ‘blunt tips and are finely serrulate’. Between 1999 and 2002, discussions on the Sea Slug Forum (hosted by the Australian Museum) between Rudman, Jensen, and others (Rudman 2001) generally supported the view that some or all of *E. abei*, *E. amakusana*, and *E. japonica* were likely synonyms, although Rudman suggested that the name *Elysia japonica* may need to be abandoned due to the lack of type material or information on the type locality. Burn (2006) restated that *E. furvacauda* was distinct from *E. japonica* which was supported by an external illustration of the former (Fig. 8), although no internal anatomy was discussed. Trowbridge et al. (2010, 2011) also discussed in detail the complexity and need for clarification between these species.

The first molecular evidence on any species in the complex was provided by Händeler and Wägele (2007), who sequenced a portion of the mitochondrial 16s rRNA for a species they called *Elysia amakusana* (from Australia) as part of a larger analysis. While this analysis provided no illustration, description, or discussion of the species in particular, an illustration of a specimen identified as *E. amakusana* from Lizard island (Australia) was published a year before by the second author (Wägele et al. 2006: 41, fig. 8H), with this specimen looking similar to *Elysia aowthai* sp. nov. A more thorough molecular assessment regarding the presently discussed species was later provided by Takano et al. (2013), where a comparison between specimens they considered representative of *E. abei*, *E. amakusana*, and E. cf. japonica was carried out. These authors provided colour photographs of *E. abei* and *E. amakusana* from Japan (Takano et al. 2013: fig. 3A, B) matching Baba´s (1949: plate VIII, fig. 26; plate IX, fig. 27) illustrations. Takano et al. (2013: fig. 3C) also provided an illustration of a specimen determined as E. cf. japonica from Japan that matches the general appearance of a species in the complex of *E. japonica*, and shows a character that could have gone unnoticed in the original description of the species due to the state of preservation of the specimens: big rounded blotches of orange pigment (different from the orange dots of *E. abei*). In their analysis, these authors concluded that *E. abei* and *E. amakusana* were likely synonyms despite their morphological variability, but E. cf. japonica appeared distinct. Takano´s et al. (2013) species identifications were based on external colouration and morphology alone. However, if the internal anatomy of their E. cf. japonica would match that of the original description, *E. japonica* could be re-described, which would help to clarify the taxonomic/systematic problems around the species complex in the area. Nonetheless, the fact is that there are at least two lineages in Japan corresponding to the external morphology of *E. abei*/*E. amakusana* and (potentially) *E. japonica*. An extensive phylogeny by Krug et al. (2015) showed specimens determined as *E. abei* (Japan), *E. amakusana* (Australia), and *E. furvacauda* (Australia) as distinct species, clustered with species they call *Elysia* sp. 5 (Hawaii) and *Elysia* sp. 30 (Japan), but again the external anatomy of the species is not shown nor discussed, which makes it impossible to draw conclusions on the validity of the different taxa.

After this review it seems clear that, within the complex, several groups may be present based on the external anatomy: one composed of specimens similar to *E. abei* /*E. amakusana* with a wide range of morphological variability, another with unclear characteristics belonging to *E. japonicasensu stricto*, and a third including *E. furvacauda*. However, the reliability of species delimitation using external colouration in this group is questionable when used alone.

On the other hand, the integrated analysis of the COI of numerous species/specimens within the complex conducted here reveals a clear geographical structure. There are at least two separate lineages in Japan (Fig. 2, clade D) corresponding to the true *E. abei*/*E. amakusana* group and (potentially) to the true *E. japonicasensu stricto* (whose type appears to be lost: Trowbridge et al. 2011). The remainder of the groups within the complex (Fig. 2, clades A–C) include *E. furvacauda* from Australia and at least two undescribed species within the range of morphological variability observed for *E. abei*/*E. amakusana* scattered in the Indo-West Pacific (I-WP).

In this work, to contribute to the untangling of the *E. japonica* complex, we describe one of these apparently widely distributed species scattered in the I-WP, *E. aowthai* sp. nov., by providing detailed morphological (external and internal) and molecular evidence. Future findings including precise sampling coordinates, complete diagnoses, colour images, and molecular data will resolve the identities of the members of this species complex and its distribution.

Despite the fact that the radular teeth of *E. aowthai* sp. nov. are indeed finely serrated, two points must be noted: these appear to be rapidly worn away with use leaving smoother edges in older teeth, and this feature is not visible under light microscopy alone and required the use of SEM observations to confirm their presence. Hence, the use of this feature alone does not have enough diagnostic significance in distinguishing the species of the complex.

*Elysia aowthai* sp. nov. is not conspecific to the specimens studied by Jensen (1985) from Hong Kong and determined as *E. japonica*: there are some differences that should be noted such as the morphology of the pericardium which is shorter, wider, and bearing four anastomosed dorsal sinuses in Jensen´s specimens, and the shape of the teeth, which are blade-shaped in *E. aowthai* sp. nov. instead of having the rounded blunt tips observed in Jensen´s specimens (Jensen 1985: fig. 2C) which appear significantly more rounded than those of *E. aowthai* sp. nov. The reproductive apparatus of Jensen´s (1985) specimens were described as ‘very similar’ to that of *Elysia verrucosa*Jensen 1985, but this statement is insufficient to establish any comparison.

## Discussion

### *Plakobranchus*

*Plakobranchus noctisstellatus* sp. nov. is externally distinctive among its currently described congeners as the only species with bright electric-blue spots under the parapodia and without a pale external colouration. Molecular evidence further supports its distinction from all other species and variants of *Plakobranchus* currently known and places it basal to the rest of the genus. The ecology of the species at its type locality also distinguishes it from *P. ocellatus* and *P. papua* which both appear to favour well-lit shallower (often intertidal) sandy habitats and coral reefs whereas *P. noctisstellatus* sp. nov. is exclusively found beyond the reef slope in deeper soft sediment habitats. There remain several aspects unknown about its biology, including its larval type and ontogenetic development. Its habitat preference probably plays an important role in its diet and its potential for (or a lack of) kleptoplasty, which is already thoroughly documented in *Plakobranchus ocellatuss. l.* (Maeda et al. 2012; Christa et al. 2013; Wade and Sherwood 2016).

The recent inventory from Koh Tao (Mehrotra and Scott 2016) documented numerous new sacoglossan records for the Gulf of Thailand. These authors were, however, unable to locate specimens of *P. noctisstellatus* sp. nov. due to its absence from shallower reefs and sandy habitats, although they were able to find and record another species of the genus which was misidentified as *Plakobranchus ianthobaptus* Gould, 1852. The same species has in fact been referred to as *Plakobranchus* sp. and Plakobranchus cf. papua by Mehrotra et al. (2015, 2019) in observations of predation. Although *Plakobranchus ianthobaptus* has long been considered a synonym of *Plakobranchus ocellatus* van Hasselt, 1824 (see Jensen 1992; Evertsen et al. 2007 and others). *Plakobranchus ocellatus* in turn has been recognised as a species complex (Christa et al. 2013; Krug et al. 2013; this paper). The recent description of *Plakobranchus papua* Meyers-Muñoz and van der Velde, 2016 included an in-depth comparison between the original descriptions of *P. ocellatus* and *P. ianthobaptus*, thus providing the clearest assessment of the latter to date (Meyers-Muñoz et al. 2016).

### *Elysia*

Utilising an integrated approach, combining molecular evidence with a wider understanding of morphological variation, we clarify the identity of a previously documented species as *Elysia aowthai* sp. nov. The wide range of external variation of the species belonging to the complex surrounding *Elysia japonica* highlights the need for a further integrative molecular and morphological investigation into externally similar specimens from Hong Kong, Guam, Australia, Hawaii, and Japan. This should also include similar and undescribed species such as those mentioned by Trowbridge et al. (2010), Takano et al. (2013), and Burn (2006). The variability in colouration is most likely driven by dietary shifts and preferences and is well documented in sea slugs (Harris 1970; Millen and Hermosillo 2012; Ekimova et al. 2017) with variation in sacoglossans usually being driven by the breakdown of ingested products (Jensen 1992; Laetz and Wägele 2018). Jensen (1992) suggested that the pigmentation of some species of *Elysia* with darker colours such as brown and purple may be due to the degradation of algal metabolites as they typically feed on plants with cellulose cell walls such as the Cladophorales.

Specimens of the *Elysia japonica* complex have been documented to be associated with the algae of the genera *Cladophoropsis* (Jensen 1993), *Chaetomorpha* (Jensen 1985; Takano et al. 2013), *Cladophora* (Takano et al. 2013), *Codium*, and *Bryopsis* (Krug et al. 2013). Interestingly, Christa et al. (2014) also identified the algae *Halimeda* sp. as prey for *Elysia amakusana*, but no information was provided on the predator specimen itself, so its precise identity is not clear. The radular morphology provided in the present description of *E. aowthai* sp. nov. does agree with the inferences of structural variation in radulae being based on diet (Jensen 1993) in turn based on the dietary trends documented for the different species in the complex. Furthermore, the discovery of some radular teeth with fine median denticulation along the cutting edge and others lacking it bridges the single difference used in Baba’s descriptions of *E. abei* and *E. amakusana* leaving only one reliable differentiating characteristic: although the tips of the teeth of *E. aowthai* sp. nov. are not really pointed, they are certainly not as rounded as those of *E. japonica*/*abei* (Fig. 8D, E). Our results do, however, indicate a possible divergence in the clade corresponding to Japanese specimens in the complex, suggesting the presence of two distinct species; therefore, a comprehensive and integrated investigation into these will be needed to assess the identity of *E. abei* and *E. amakusana*. This further supports the need for a closer investigation on the role of dietary plasticity on radular morphology within the *Elysia japonica* complex. Recent findings have also shown that the specimens of *E. aowthai* sp. nov. from Koh Tao are relatively unpalatable to potential scleractinian predators, which may be driven by the toxicity sequestered from their prey (Mehrotra et al. 2019).

## Conclusions

The integration of ecological data, with morphology, ontogeny, and molecular evidence will contribute towards addressing the cryptic species problem increasingly striking in heterobranch sea slugs systematics (Korshunova et al. 2019). Within the Plakobranchidae, the difference in diversity between accepted species (corresponding to external variation) and those suggested by molecular evidence continues to grow. Within *Plakobranchus*, our analyses and those of Krug et al. (2013) suggest that the presently described and delineated species comprise only a third of the possible diversity indicated by molecular means. *Elysia* has also recently been shown to contain several species complexes that require a closer examination, such as *Elysia marginata*, *E. pusilla*, and *E. tomentosa* (Krug et al. 2013, 2016; Yonow and Jensen 2018). As with *Plakobranchus ocellatus* and *Elysia marginata*, evidence that the synonymisation of multiple species and morphotypes under a single name may have been premature has been documented for *Thuridilla gracilis* (Yonow and Jensen 2018; Papu et al. 2020).

Both species documented in this work, *P. noctisstellatus* sp. nov. and *E. aowthai* sp. nov., had been considered unknown or locally rare from Koh Tao until expansion of surveys into deeper soft sediment habitats. Exploration of these same habitats have recently allowed for documentation of previously undescribed species and ecology from Koh Tao (Mehrotra et al. 2017, 2019). Investigation of ecological parameters such as habitat preferences, dietary plasticity, and other interspecies interactions should be included in future attempts studies of species complexes, as they may contribute significantly in the clarification of the cryptic species problems highlighted here.

## Supplementary Material

XML Treatment for Plakobranchus

XML Treatment for Plakobranchus ocellatus

XML Treatment for Plakobranchus papua

XML Treatment for Plakobranchus noctisstellatus

XML Treatment for Elysia

XML Treatment for Elysia aowthai

## Supplementary materials

Supplementary material 1 *Plakobranchus*COI topology difference Data type Phylogeny Explanation note *Plakobranchus*COI topology difference – Maximum Likelihood (left) and Bayesian Inference (right) phylogenetic analyses of the cytochrome oxidase 1 gene provide very similar topologies. Differences in node and branch placement are here highlighted using Fig. 3 as a baseline for comparison. https://binary.pensoft.net/file/454254This dataset is made available under the Open Database License (http://opendatacommons.org/licenses/odbl/1.0/). The Open Database License (ODbL) is a license agreement intended to allow users to freely share, modify, and use this Dataset while maintaining this same freedom for others, provided that the original source and author(s) are credited.Rahul Mehrotra, Manuel Caballer Gutiérrez, Chad M. Scott, Spencer Arnold, Coline Monchanin, Suchana Chavanich

Supplementary material 2 *Plakobranchus ocellatus* mating Data type multimedia Explanation note *Plakobranchus ocellatus* mating – Two individuals of *Plakobranchus ocellatus* at Koh Tao, Thailand, exhibit trailing and mating behaviour. https://binary.pensoft.net/file/454255This dataset is made available under the Open Database License (http://opendatacommons.org/licenses/odbl/1.0/). The Open Database License (ODbL) is a license agreement intended to allow users to freely share, modify, and use this Dataset while maintaining this same freedom for others, provided that the original source and author(s) are credited.Rahul Mehrotra, Manuel Caballer Gutiérrez, Chad M. Scott, Spencer Arnold, Coline Monchanin, Suchana Chavanich

Supplementary material 3 *Plakobranchus papua* mating Data type multimedia Explanation note *Plakobranchus papua* mating – Two individuals of *Plakobranchus papua* at Koh Tao, Thailand, exhibit trailing and mating behaviour. https://binary.pensoft.net/file/454256This dataset is made available under the Open Database License (http://opendatacommons.org/licenses/odbl/1.0/). The Open Database License (ODbL) is a license agreement intended to allow users to freely share, modify, and use this Dataset while maintaining this same freedom for others, provided that the original source and author(s) are credited.Rahul Mehrotra, Manuel Caballer Gutiérrez, Chad M. Scott, Spencer Arnold, Coline Monchanin, Suchana Chavanich
