# Supplementary figures and images for: On the Plakobranchidae (Gastropoda, Sacoglossa) from soft sediment habitats of Koh Tao, Gulf of Thailand, with descriptions of two new species
Source: Zookeys. 2020 Sep 17;969:85–121. doi: 10.3897/zookeys.969.52941 (PMC7515966; doi:10.3897/zookeys.969.52941)

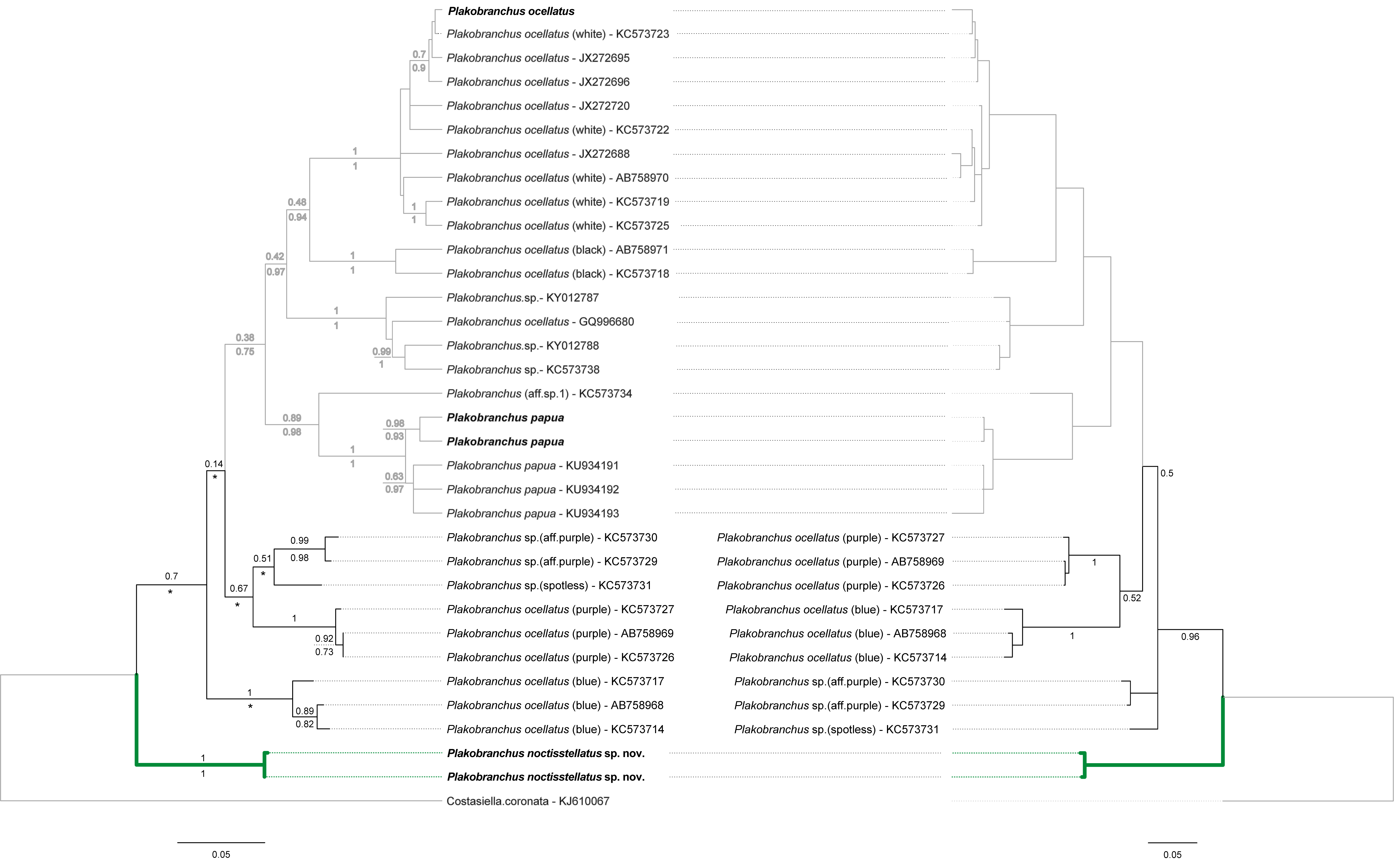

Supplement: Supplementary material 1 — PlakobranchusCOI topology difference [file zookeys-969-085-s001.jpg]
